# A Case of Adenoid Cystic Carcinoma Mimicking a Bartholin Cyst and Literature Review

**DOI:** 10.1155/2018/5256876

**Published:** 2018-03-26

**Authors:** Siew Pei Goh, Brian McCully, Matthew Kyle Wagner

**Affiliations:** ^1^Ipswich Clinical Unit, University of Queensland, 7 Chelmsford Avenue, Ipswich, QLD 4305, Australia; ^2^Obstetrics and Gynaecology, Ipswich General Hospital, 7 Chelmsford Avenue, Ipswich, QLD 4305, Australia; ^3^University of Queensland, Brisbane, QLD, Australia

## Abstract

Adenoid cystic carcinoma of the Bartholin Gland (BG-ACC) is a rare form of vulval cancer with only approximately 350 reported cases since 1864. A review of available literature and case reports suggests an aggressive nature with protracted clinical symptoms and a tendency for local recurrence despite adequate surgical excision with or without adjuvant radiotherapy. Survival rates of 71% and 59% are reported at five and ten years. This case report endeavours to add to our body of knowledge regarding this rare disease and thus help broaden and improve our understanding of management and treatment success.

## 1. Introduction

We report a case of BG-ACC in Ipswich General Hospital, QLD.

## 2. Case Presentation

A 41-year-old slim female with no previous gynaecological history was seen in the Ipswich Gynaecology Clinic with a 3-month history of a small 2 cm painless swelling thought to be a left sided Bartholin Gland (BG) cyst. There was no significant past medical history. The patient was booked for elective marsupialisation. At the time of surgery, a solitary solid and mobile mass was noted in the BG, extending into subcutaneous and muscle tissue. An excisional biopsy was performed. Histology identified changes consistent with Adenoid Cystic Carcinoma of the BG involving surgical margins with perineural invasion. The tumour showed extensive cribriform and tubular islands of malignant epithelium infiltrating hyaline stroma, containing intraluminal basement membrane like material. It also stained positive with Calponin and p53, markers which are also found in adenoid cystic carcinoma of the salivary gland. A staging CT of the chest, abdomen, and pelvis revealed no evidence of metastasis. Patient underwent radical local incision and bilateral inguinal lymph node dissection and was diagnosed with FIGO Stage 1b Vulval cancer. She completed six cycles of adjuvant radiotherapy, 63 Gy/33 fractions to her vulva using VMAT (Vaginal Sparing with Volumetric Modulated Arc Therapy), and has remained disease-free at 9 months with preservation of sexual function.

## 3. Discussion

Adenoid cystic carcinoma (ACC) of the Bartholin Gland (BG) is a rare form of vulval cancer with only approximately 350 reported cases since Klob in 1864 [1].

ACC constitutes 15–25% of all Bartholin Gland cancers.

The average age of diagnosis is 49 years with range between 25 and 80 years [2].

To date, little is known regarding the natural history of ACC of BG. It most often presents without antecedent history of benign disease. Once diagnosed, its behaviour is often described as biologically aggressive and unpredictable. Its clinical course is often protracted and there is a tendency for perineural invasion and local recurrence [2, 3]. Felix et al. 1993 suggested that Human papillomavirus may play a pathogenic role [4]. Copeland et al. 1986 identified a possible link with pregnancy, noting that 7 of 14 of their patients were pregnant at the time of diagnosis. In one patient, growth of the tumour was rapid suggesting hormonal sensitivity [3].

BG-ACC can present as a vulval mass, with or without symptoms such as pain, ulceration, pruritus, abnormal bleeding, or dyspareunia. It may also present as palpable solid, cystic, or abscessed area within a Bartholin cyst. The mass rarely occurs bilaterally. Clinicians should have heightened suspicion for malignancy when there is palpable solid mass within the cyst, underlying fixation to surrounding tissues, and/or cyst or abscess that does not respond or worsens despite treatment [5]. Due to its tendency to infiltrate perineural spaces, patients may experience itching and burning before a mass becomes palpable [6].

ACC is a histological diagnosis. Bartholin Gland ACC is histologically similar to ACC of the salivary gland, upper respiratory tract (lacrimal, nasopharynx), and breast. There are 3 histological types of ACC, that is, cribriform, tubular, and solid. Of these, the tubular form tends to be best differentiated and confers the most favourable prognosis. On the other hand, solid form is associated with highest incidence of metastasis and significantly lower survival rate [7, 8].

Microscopic examination of BG-ACC typically demonstrates cribriform pattern consisting of nests and columns of cells arranged concentrically around gland-like spaces filled with eosinophilic Periodic Acid-Schiff- (PAS-) Positive material [9–12]. Immunohistochemical staining may reveal low-molecular-weight keratins, carcinoembryonic antigen, lysozyme, alpha-antichymotrypsin, S100, and type IV collagen [11]. Ramanah et al. took a step further by performing specific antibody stains of brain metastases from BG-ACC, which revealed positive cytokeratin (CK) AE1/AE3, CK7, and epithelial membrane antigen (EMA); weakly positive S100 and progesterone receptor; and negative CK20 and estrogen receptor [10].

Initial treatment of Bartholin Gland Cancer is similar as that for vulval cancer. Literature review supports wide local excision, with or without inguinal lymphadenectomy [13, 14]. Post-op adjuvant radiotherapy is generally recommended when there is a positive surgical margin, deep local and perineural invasion, and local recurrence [15, 16]. Data is lacking for the role of chemotherapy and biological treatment of BG-ACC. Few studies have trialled 5-fluorouracil, adriamycin, cyclophosphamide, cisplatin, CPT-11 (Irinotecan), methotrexate, and doxorubicin with varying efficacy [17–20]. A single patient trial of adjuvant Tamoxifen on metastatic BG-ACC delayed disease progression for 4 years while maintaining quality of life [21].

Prognosis for BG-ACC is similar to a low grade vulval cancer. Survival was quoted by Lelle et al. 1994 to exceed 71% and 59% at five and ten years [2, 19]; while disease-free rates were reported to be 47–83% and 33–38%, respectively [11]. The longest reported survival is 31 years [2].

Surgical resection margin appears to be the single most important factor for cure [22]. Groin nodal status on the other hand is thought to be the main determinant of survival [23]. Routine lymphadenectomy however does not appear to improve survival and prognosis [20]. Postoperative adjuvant radiotherapy is thought to be effective in reducing recurrence rate for positive surgical margins and deep local and perineural invasion [18].

Sadly, local recurrence and metastasis can occur long after primary treatment [24]. Bones and lungs are the most common site of metastasis; other sites reported are liver, kidney, and brain [25]. Late recurrences and metastases are associated with poor prognosis [9, 10].

More research and case reports on this rare cancer are needed in order to further comprehend and manage the disease. The role of chemotherapy and biological treatment with hormones or receptor antibody has potential for controlling local recurrence and metastatic disease of adenoid cystic carcinoma of the vulva and their role should be further explored.

The purpose of this report is to remind the clinical practitioner that Adenoid cystic carcinoma (ACC) of the Bartholin Gland (BG) can occur. The authors acknowledge its rarity but suggest that a differential diagnosis which includes a suspicion of its role in the aetiology of Bartholin Gland disorders may well help the optimization of appropriate patient care.

## Supplementary Materials

Supplementary Materials (1) Contrast CT showing an irregular area on the left perineum consistent with recent biopsy. (2) H&E stain of the tumour showing (A) perineural invasion and (B) cribriform-tubular arrangement of tumour cells around acellular spaces and pseudomucinous basement membrane.
