# Supplementary material for: A Case of Adenoid Cystic Carcinoma Mimicking a Bartholin Cyst and Literature Review
Source: Case Rep Obstet Gynecol. 2018 Mar 26;2018:5256876. doi: 10.1155/2018/5256876 (PMC5892282; doi:10.1155/2018/5256876)

**2B) Cribriform-tubular arrangement of tumour cells around acellular spaces and pseudo-mucinous basement membrane. H&E stain**


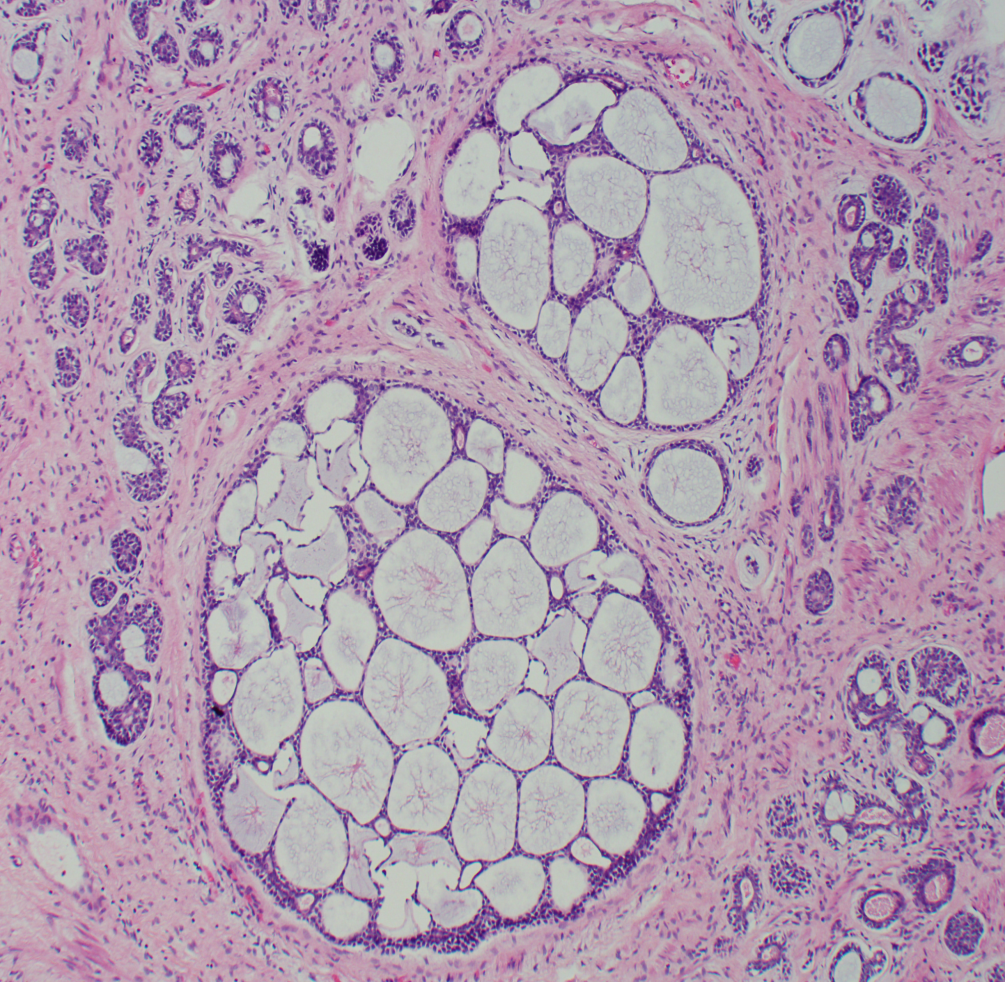

Supplement: Supplementary Materials — (1) Contrast CT showing an irregular area on the left perineum consistent with recent biopsy. (2) H&E stain of the tumour showing (A) perineural invasion and (B) cribriform-tubular arrangement of tumour cells around acellular spaces and pseudomucinous basement membrane. [file 5256876.f1.zip › 5256876.f1/path slide 2_CRIOG_2207410.docx]
